# Disaster-Related Digital Technology Engagement and Preparedness Beliefs Among Primary Care Attendees: A Health Belief Model-Based Cross-Sectional Study in Türkiye

**DOI:** 10.3390/healthcare14142232

**Published:** 2026-07-22

**Authors:** Ebru Uğraş, Safiye Kübra Çetindağ Karatlı, Erhan Şimşek, Öykü Su Tulumtaş, Melike Yeşil, Ahmet Keskin

**Affiliations:** 1 Department of Family Medicine, Ankara Yıldırım Beyazıt University, Ankara 06220, Türkiye; 2 Department of Family Medicine, Ankara Bilkent City Hospital, Ankara 06800, Türkiye; 3 General Directorate of Public Health, Republic of Türkiye Ministry of Health, Ankara 06100, Türkiye; 4 Faculty of Medicine, Ankara Yıldırım Beyazıt University, Ankara 06220, Türkiye; melikeyesilny@gmail.com

**Keywords:** disaster preparedness, digital health technologies, primary care, health belief model, risk communication, healthcare resilience, public health preparedness

## Abstract

**Highlights:**

**What are the main findings?**

- Disaster-related digital technology engagement remained independently associated with overall disaster preparedness beliefs after adjustment for age, gender, education, income, marital status, and occupation.
- Adjusted associations were observed for perceived susceptibility, perceived benefits, perceived low barriers, cues to action, and self-efficacy, but not for perceived severity.

**What are the implications of the main findings?**

- Primary care services can support preparedness through official alert applications, emergency-notification guidance, digital information-verification skills, and community-based education.
- Digital interventions should be combined with non-digital alternatives and targeted digital-literacy support to avoid widening socioeconomic inequalities.

**Abstract:**

**Background/Objectives:** Disaster preparedness is central to public health resilience. This study examined the association between disaster-related digital technology engagement and Health Belief Model-based preparedness beliefs among adults attending a primary care clinic in Türkiye. **Methods:** In this cross-sectional study, 538 participants completed a sociodemographic form, a researcher-developed 10-item Disaster and Technology Use Questionnaire, and the 31-item General Disaster Preparedness Belief Scale. The technology questionnaire used five-point Likert-type response categories and was treated as a multidomain questionnaire; its prespecified equal-weighted sum formed an operational composite index rather than a unidimensional scale score. Supplementary internal-structure analyses described item covariance and clustering. Spearman correlations, false-discovery-rate correction, and hierarchical multiple linear regression with HC3 robust standard errors were used, adjusting for age, gender, education, income, marital status, and occupation. **Results:** The technology composite showed α = 0.793 and ω = 0.799, and exploratory and confirmatory analyses identified two correlated empirical item domains with adequate model fit (CFI = 0.952, TLI = 0.936, RMSEA = 0.055). Technology engagement was independently associated with total preparedness beliefs (B = 0.888, 95% CI 0.672–1.103; standardized β = 0.397; *p* < 0.001) and increased explained variance by 13.3% beyond sociodemographic factors. Adjusted associations remained significant for perceived susceptibility, perceived benefits, perceived low barriers, cues to action, and self-efficacy, but not for perceived severity. **Conclusions:** Greater disaster-related digital technology engagement was associated with stronger preparedness beliefs, but causal direction cannot be inferred. Primary care initiatives may promote official alert tools, practical application use, and digital-information literacy while maintaining non-digital alternatives for people with limited digital access.

## 1. Introduction

Disasters are complex events that significantly disrupt human life, health systems, and socioeconomic structures. In recent decades, both the frequency and severity of disasters have increased globally due to factors such as climate change, rapid urbanization, and infrastructural vulnerabilities [1,2]. This upward trend underscores the importance of disaster preparedness as a key strategy to reduce disaster-related losses and improve community resilience [3,4].

Individual disaster preparedness is a multidimensional process shaped by risk perception, knowledge, previous experience, and environmental factors. Risk perception, defined as an individual’s evaluation of the likelihood and potential consequences of a hazard, is one of the most important psychological determinants of protective behavior [5,6,7]. Studies have consistently demonstrated that individuals with higher perceived risk are more likely to adopt preparedness behaviors in various disaster contexts, including earthquakes, floods, and fires [8]. In addition, self-efficacy and perceived benefits have also been identified as critical associated factors of preparedness behaviors, influencing individuals’ motivation to take preventive actions [9,10].

The Health Belief Model (HBM) explains protective behavior through six interrelated constructs. Perceived susceptibility refers to an individual’s belief about the likelihood of experiencing a hazard, whereas perceived severity reflects the expected seriousness of its consequences. Perceived benefits concern the expected value of preparedness actions, and perceived barriers represent the practical, financial, cognitive, or social obstacles to those actions. Cues to action are internal or external triggers—such as warnings, education, media messages, or advice from healthcare professionals—that prompt preparedness. Self-efficacy reflects confidence in one’s ability to carry out the required actions [11,12]. In a disaster context, these constructs help explain why awareness does not automatically translate into preparedness and why information, capability, and actionable prompts must be considered together.

Sociodemographic factors (age, gender, education, and income) shape disaster preparedness by influencing risk perception and preparedness levels [13]. Prior disaster experience further increases awareness and preparedness intentions [14]. However, preparedness cannot be explained by awareness alone; it requires the integration of cognitive, behavioral, and environmental supports. Education, communication strategies, and drills act as key cues to action [9], while perceived barriers, such as time constraints, financial limitations, and lack of knowledge, can hinder preparedness [10].

In recent years, digital technologies have played an increasingly important role in disaster preparedness and management. Tools such as early warning systems, mobile applications, and social media support risk communication and emergency response [3,4,15,16,17]. They enable rapid information sharing and improve access to critical resources, enhancing preparedness and response efficiency [3]. However, these platforms also pose challenges, including misinformation and poor decision-making, which may undermine preparedness [3]. Therefore, digital literacy and access to reliable information are essential for effective disaster preparedness.

Recent evidence suggests that communities with stronger digital communication infrastructures and higher levels of technological engagement tend to demonstrate improved preparedness outcomes [2,3]. Nevertheless, the effectiveness of these technological tools depends largely on individuals’ digital literacy, trust in technology, and their ability to integrate digital information into decision-making processes [4]. Despite these developments, there remains a limited number of studies examining the relationship between disaster-related digital technology use and preparedness beliefs within a comprehensive behavioral framework.

Overall, disaster preparedness should be understood as a dynamic interaction between social, individual, psychological, and technological factors. Examining these components within an integrated framework is essential for developing evidence-based disaster risk reduction strategies and strengthening public health resilience [3,4].

**Figure 1:**
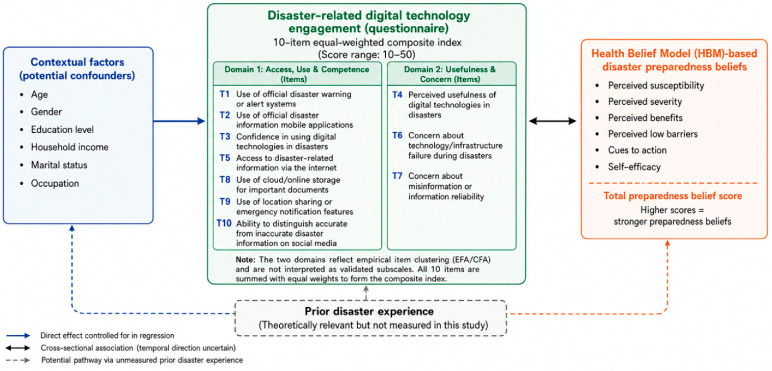
Conceptual framework linking contextual factors, disaster-related digital technology engagement, and Health Belief Model-based preparedness beliefs. Note: T1–T10 represent the questionnaire items included in the equal-weighted composite index. The two empirical domains identified by EFA/CFA are not interpreted as validated subscales. The double-headed arrow indicates uncertain temporal direction due to the cross-sectional design. Prior disaster experience was theoretically relevant but not measured.

For the present study, the Disaster and Technology Use Questionnaire was not designed as a psychometric scale intended to measure a single reflective latent trait. Rather, it was designed as a multidomain questionnaire covering complementary and non-interchangeable components of disaster-related digital engagement: access to and use of warning applications and digital resources, perceived technical competence, access to accurate information, information-verification ability, perceived usefulness, and concern about technological failure. A prespecified sum of the 10 Likert-type item responses was used as an operational composite index [18,19]. These components were combined because effective digital preparedness requires not only possession or use of technology, but also the ability to access, interpret, and act on information while recognizing infrastructure vulnerabilities. The proposed conceptual framework is presented in Figure 1.

Therefore, the primary aim of this study was to examine whether disaster-related digital technology engagement, operationalized using the prespecified questionnaire-derived composite index, was independently associated with disaster preparedness beliefs among adults attending a family medicine outpatient clinic in Türkiye. Secondary aims were to describe the empirical internal structure and clustering of the questionnaire items and to identify which HBM dimensions remained associated with technology engagement after adjustment for sociodemographic factors.

## 2. Materials and Methods

### 2.1. Study Design and Sample

This cross-sectional analytical study was conducted in a family medicine outpatient clinic in Ankara, Türkiye. A convenience sampling approach was used: adults aged 18 years or older who attended the clinic during the data-collection period and voluntarily agreed to participate were eligible. Individuals with incomplete questionnaires or communication difficulties were excluded. Because recruitment was performed in a single clinic without probability sampling, national weighting, or population-based sampling frames, the sample represents participating primary care attendees rather than the general adult population of Türkiye. An a priori sample-size calculation was performed using G*Power version 3.1 for a two-tailed comparison of two independent means. The calculation assumed a two-sided alpha level of 0.05 (corresponding to a 95% confidence level), 80% statistical power, a small-to-moderate standardized effect size of Cohen’s d = 0.30, and an allocation ratio of 1:1 [20,21]. For transparency, the conventional normal-approximation equation corresponding to this calculation was:

$$n_{group}=\frac{2{\left(Z_{1-\alpha /2}+Z_{1-\beta }\right)}^{2}}{d^{2}}$$

where *ngroup* is the required number of participants in each group, *Z*1 − *α*/2 is the critical value corresponding to the two-sided significance level, *Z*1 − *β* is the critical value corresponding to the desired statistical power, and *d* is the standardized mean difference. The total sample size was defined as:

$$N=2n_{group}$$

After conservative upward rounding to account for potentially incomplete or invalid questionnaires, the minimum recruitment target was set at 360 participants. A total of 538 participants with complete data were included in the analysis.

### 2.2. Data Collection Instruments

Data were collected using a structured questionnaire consisting of three parts: (1) a sociodemographic information form, (2) the researcher-developed Disaster and Technology Use Questionnaire, and (3) the General Disaster Preparedness Belief Scale (GDPBS).

The Disaster and Technology Use Questionnaire consisted of 10 items and was developed as a multidomain questionnaire rather than as a unidimensional psychometric scale. The items intentionally assessed complementary and non-interchangeable components of disaster-related digital engagement: knowledge of official disaster applications and warning systems; active use of emergency notifications; availability of tools such as power banks and radios; perceived importance of digital maps and location sharing; perceived technical competence; concern about technological failure; perceived usefulness of technology in post-disaster aid; access to accurate digital information; digital storage of important documents; and the ability to distinguish accurate from inaccurate disaster information on social media. Each item used five-point Likert-type response categories ranging from 1 = strongly disagree to 5 = strongly agree. The technology-failure item was retained because awareness of infrastructure vulnerability was considered a relevant component of practical digital preparedness. As prespecified before analysis, the 10 responses were summed with equal weighting to create an operational composite index ranging from 10 to 50; higher values indicated broader disaster-related digital engagement and preparedness-related digital awareness across the included domains. Because the items were not assumed to be parallel manifestations of a single latent trait, the composite was not interpreted as a validated scale score or diagnostic measure [18,19]. Items were generated from the disaster-preparedness and digital-technology literature and pilot-tested in 20 adults for clarity and comprehensibility. No formal quantitative content-validity index or external expert-panel assessment was conducted before data collection. Cronbach’s alpha, McDonald’s omega, item-total correlations, EFA, and CFA were therefore used only as supplementary descriptive analyses of item covariance and empirical clustering in the present sample; they were not interpreted as establishing full psychometric validation or unidimensionality.

The General Disaster Preparedness Belief Scale (GDPBS), developed and validated in Turkish by Inal et al. [11], was used to assess HBM-based disaster preparedness beliefs. The 31 items are organized into perceived susceptibility (items 1–6), perceived severity (items 7–9), perceived benefits (items 10–12), perceived low barriers (items 13–18), cues to action (items 19–23), and self-efficacy (items 24–31). Each item is rated from 1 = strongly disagree to 5 = strongly agree. Negatively worded responses were reverse-coded during data entry, as confirmed from the original SPSS (IBM SPSS Statistics for Windows, Version 31.0; IBM Corp., Armonk, NY, USA) syntax, so that higher values consistently represented stronger preparedness beliefs; specifically, a higher perceived-low-barriers score indicates fewer perceived barriers. The six subscale scores were summed to obtain a total score ranging from 31 to 155, with no diagnostic cut-off. In the original validation study, the scale explained 59.2% of the variance, subscale alpha coefficients ranged from 0.74 to 0.90, and the stratified alpha for the total scale was 0.93 [11]. In the present sample, both Cronbach’s alpha and McDonald’s omega were calculated for the total scale and each subscale.

### 2.3. Data Collection Procedure

Data were collected through face-to-face interviews with participants who provided informed consent to take part in the study. Before data collection, participants were informed about the purpose of the study, and informed consent was obtained. Data were collected between March and May 2026.

### 2.4. Statistical Analysis

Descriptive and inferential analyses were performed using IBM SPSS Statistics and independently reproduced in Python 3.11 using statsmodels, factor_analyzer, and semopy. Continuous composite and scale scores were summarized using medians and interquartile ranges because they were not normally distributed. For the GDPBS, internal consistency was evaluated using Cronbach’s alpha and McDonald’s omega. For the researcher-developed technology questionnaire, alpha, omega, corrected item-total correlations, and alpha-if-item-deleted values were examined as supplementary descriptions of item covariance; because the instrument was conceptualized as a multidomain composite, these coefficients were not treated as evidence of unidimensional scale reliability. Sampling adequacy for exploratory analysis of the questionnaire items was evaluated using the Kaiser–Meyer–Olkin statistic and Bartlett’s test of sphericity. The sample was randomly divided, using a fixed random seed, into an EFA subset (*n* = 269) and an independent CFA subset (*n* = 269). EFA used minimum residual extraction with oblimin rotation, and the number of empirical item domains was determined by 1000-replication parallel analysis. CFA was estimated using diagonally weighted least squares; chi-square, CFI, TLI, and RMSEA were evaluated jointly. These analyses were used to describe item clustering and were not interpreted as formal validation of a unidimensional scale or as establishing validated subscales. Spearman’s rho was used for bivariate associations, with 95% confidence intervals calculated by Fisher’s z transformation. Mann–Whitney U and Kruskal–Wallis tests were used for group comparisons, accompanied by rank-biserial correlations or epsilon-squared effect sizes. Benjamini–Hochberg false discovery rate (FDR) adjustment was applied within related families of secondary comparisons [22]. To estimate the independent association between the technology composite index and preparedness beliefs, hierarchical multiple linear regression was performed. Age, gender, education, income, marital status, and occupation were entered in Model 1, followed by the technology composite index in Model 2. The same covariates were used in separate secondary models for the six HBM subscales. Because the Breusch–Pagan test indicated mild heteroscedasticity, HC3 robust standard errors and 95% confidence intervals were reported. Multicollinearity, residual distribution, model specification, and influential observations were assessed using variance inflation factors, the Jarque–Bera test, the Ramsey RESET test, and Cook’s distance. Statistical significance was set at *p* < 0.05.

### 2.5. Ethical Approval

Ethical approval was obtained from the Ankara Bilkent City Hospital 2nd Clinical Research Ethics Committee (Decision No. TABED 2/1934/2026; official document No. TABED 2-26-1934; 4 February 2026). The study was conducted in accordance with the Declaration of Helsinki.

## 3. Results

A total of 538 individuals participated in the study. The sociodemographic characteristics of the participants are presented in Table 1.

Descriptive statistics and internal-consistency coefficients are summarized in Table 2. The 10-item questionnaire-derived technology engagement composite index had Cronbach’s α = 0.793 and McDonald’s ω = 0.799; these coefficients are reported as descriptive indicators of item covariance rather than as proof that the questionnaire is a unidimensional scale. The GDPBS total score showed good internal consistency (α = 0.866; ω = 0.876). Perceived benefits had the highest reliability. Perceived susceptibility showed borderline internal consistency (α = 0.599; ω = 0.624), indicating that findings involving this subscale require cautious interpretation.

The technology-item correlation matrix was suitable for exploratory analysis (Kaiser–Meyer–Olkin = 0.819; Bartlett χ^2^(45) = 1311.50, *p* < 0.001). Parallel analysis identified two correlated empirical item domains. In the EFA subset, the first domain comprised access, use, and perceived competence items, whereas the second comprised perceived value and technology-related concern items (Table 3). The corresponding two-domain CFA showed adequate overall fit (χ^2^(34) = 61.87, *p* = 0.002; CFI = 0.952; TLI = 0.936; RMSEA = 0.055), with a domain correlation of 0.574. Alpha coefficients for the two item clusters were 0.770 and 0.661, respectively. Convergent-structure indices were modest (composite reliability/average variance extracted: 0.727/0.279 for Domain 1 and 0.659/0.406 for Domain 2), and item T6 had the lowest corrected item-total correlation (r = 0.258); Cronbach’s alpha would be 0.798 if this item were deleted. This finding is consistent with T6’s distinct focus on technology-failure concern. Because the questionnaire was intentionally multidomain, these analyses were interpreted as descriptions of empirical item clustering rather than as evidence of a single latent construct or formally validated subscales. The prespecified composite index was retained as the primary operational summary; sensitivity analyses using the two empirical domain scores produced associations in the same direction.

Bivariate associations are presented in Table 4. Disaster-related technology engagement was positively associated with total preparedness beliefs (Spearman’s ρ = 0.388, 95% CI 0.313–0.457, *p* < 0.001). After FDR correction, significant associations remained with perceived susceptibility, perceived benefits, perceived low barriers, cues to action, and self-efficacy, whereas perceived severity was not associated with technology engagement.

Gender comparisons are shown in Table 5. After FDR correction, women had higher total preparedness belief, perceived susceptibility, perceived severity, and perceived-low-barriers scores than men. The previously nominal difference in cues to action did not remain significant after FDR correction (q = 0.055). Technology engagement, perceived benefits, and self-efficacy did not differ significantly by gender. Effect sizes were small.

Education and income comparisons are presented in Table 6 and Table 7. Technology engagement and total preparedness beliefs differed across education groups after FDR correction, with small-to-moderate overall effects. Post hoc FDR-adjusted comparisons showed that university-educated participants had higher technology scores than both lower-education groups and higher preparedness scores than both groups; the preparedness difference between primary/secondary and high-school groups was not significant. Participants with income greater than expenses also had higher technology and preparedness scores, with small effect sizes.

Hierarchical regression results are presented in Table 8. Sociodemographic variables explained 14.8% of the variance in total preparedness beliefs. Adding technology engagement increased explained variance by 13.3% (ΔR^2^ = 0.133, *p* < 0.001), resulting in a final R^2^ of 0.281 and adjusted R^2^ of 0.262. Technology engagement remained independently associated with total preparedness beliefs (B = 0.888 per one-point increase, HC3 95% CI 0.672–1.103; standardized β = 0.397; *p* < 0.001). The incremental effect size was Cohen’s f^2^ = 0.186, indicating a practically meaningful small-to-moderate contribution. The technology predictor showed no concerning multicollinearity (VIF = 1.18), and residual diagnostics did not indicate major non-normality, misspecification, or influential observations.

Adjusted associations with the HBM subscales are shown in Table 9. After covariate adjustment and FDR correction, the technology composite index remained associated with perceived susceptibility, perceived benefits, perceived low barriers, cues to action, and self-efficacy. The adjusted association with perceived severity remained non-significant. In a sensitivity analysis replacing the total composite index with the two empirical domain scores, both access/use/competence (B = 0.738, standardized β = 0.264, *p* < 0.001) and perceived value/technology-related concerns (B = 1.396, standardized β = 0.219, *p* < 0.001) were independently associated with total preparedness beliefs.

## 4. Discussion

This study provides primary care-based evidence that disaster-related digital technology engagement is independently associated with HBM-based preparedness beliefs in a disaster-prone setting. The association remained after adjustment for age, gender, education, income, marital status, and occupation. Technology engagement contributed an additional 13.3% of explained variance beyond these sociodemographic factors, with a standardized coefficient of 0.397 and Cohen’s f^2^ of 0.186. Thus, the relationship was not merely statistically significant; it was of modest-to-moderate practical magnitude. Nevertheless, most variance remained unexplained, confirming that technology is only one component of the wider social, psychological, and structural determinants of preparedness.

The adjusted associations with perceived susceptibility, perceived benefits, perceived low barriers, self-efficacy, and cues to action are consistent with the HBM. Digital alerts and official applications may operate as cues to action; reliable instructions and location-sharing tools may increase perceived benefits and self-efficacy; and accessible information may reduce knowledge-related barriers [11,12,23]. The largest adjusted subscale association was observed for cues to action, supporting the view that digital technologies are especially relevant as prompts and channels for actionable preparedness information. Because the effect sizes were not large, digital tools should complement rather than replace community education, drills, social support, and structural preparedness measures.

Perceived severity was not associated with technology engagement in either bivariate or adjusted analyses. This is consistent with evidence that severity can be a weaker or less consistent predictor than benefits, barriers, and self-efficacy [12,24]. Perceptions of disaster seriousness may be influenced more strongly by direct experience, cultural context, emotional responses, and the perceived consequences for family and livelihood than by digital engagement alone [25]. Digital information may therefore be more effective at supporting action-oriented beliefs than at changing views about the magnitude of disaster consequences.

The internal-structure analyses clarify the technology measure raised by the reviewers. The Disaster and Technology Use Questionnaire was not intended to function as a conventional unidimensional psychometric scale. Its items represent complementary components that together form a prespecified operational composite index [18,19]. The exploratory analyses identified two correlated empirical item domains—access/use/competence and perceived value/technology-related concerns—and the corresponding two-domain CFA showed adequate global fit. These domains are presented as descriptive item clusters, not as formally validated subscales. Likewise, alpha and omega are reported as supplementary indicators of item covariance rather than as proof of unidimensionality. The modest convergent-validity indices, the comparatively weak item-total correlation for the technology-failure concern item, equal weighting of heterogeneous components, and absence of formal content and external validation all support cautious interpretation. Accordingly, findings should be understood as associations involving a researcher-defined questionnaire-derived composite index that requires refinement and replication in independent populations.

Women had higher total preparedness, susceptibility, severity, and perceived-low-barriers scores after FDR correction, although the effect sizes were small. Technology engagement did not differ by gender. Education and income showed clearer gradients: university-educated and higher-income participants generally had higher technology and preparedness scores. The sample also differed from national demographic patterns. Women represented 55.8% of the study sample compared with 49.98% of Türkiye’s population in 2025 [26]. University-level education or higher was reported by 68.4% of participants, whereas tertiary education was reported for 25.3% of Türkiye’s population aged 25 years or older in 2024 [27]; this comparison is approximate because the age denominators differ. The high proportion of university graduates and civil servants likely reflects the single-clinic Ankara setting and limits generalizability.

These socioeconomic patterns emphasize the risk of a digital preparedness divide. Higher education can facilitate the interpretation and verification of risk information, while higher income can support access to smartphones, connectivity, backup power, and other resources [28,29]. Digital preparedness policies should therefore include targeted support for people with limited access, lower digital literacy, older devices, or intermittent connectivity. Maintaining non-digital warning channels, printed materials, radio communication, and face-to-face instruction remains essential.

The findings also have practical implications for risk communication. Primary care teams could incorporate brief guidance on activating official emergency alerts, installing and using verified disaster applications, using location-sharing and offline maps, storing essential documents securely, and identifying official information sources. Short digital-literacy modules could teach patients how to recognize misinformation and verify disaster-related messages. Community-based education sessions, especially for lower-income and lower-education groups, could combine hands-on application practice with conventional preparedness training. These interventions should be co-designed for accessibility and should not assume continuous electricity, mobile coverage, or internet access [16,30].

The findings should also be interpreted within Türkiye’s disaster-risk context. Türkiye is highly exposed to earthquakes and other natural hazards, and the 2023 Kahramanmaraş earthquakes demonstrated the need to strengthen community preparedness, risk communication, and healthcare-system resilience [31,32,33]. Family medicine clinics are accessible settings for directing patients to official warning systems, identifying individuals with low preparedness capacity, and linking digital information with practical household planning. However, the cross-sectional association cannot establish whether technology engagement strengthens preparedness beliefs. Reverse causation is equally plausible: people with stronger preparedness beliefs, greater self-efficacy, or more cues to action may be more likely to seek and use disaster-related technologies. Longitudinal and intervention studies are needed to establish temporal direction and determine whether digital interventions improve observed preparedness behavior.

Overall, disaster preparedness is a multidimensional process shaped by the interaction of individual beliefs, social resources, prior experiences, technology, healthcare access, and structural conditions. Digital tools may increase reach and timeliness, but they cannot compensate for weak infrastructure, unequal access, or the absence of community-level preparedness programs. Comprehensive strategies should therefore integrate digital communication with primary care counseling, community drills, social support, and inclusive non-digital alternatives.

## 5. Strengths and Limitations

This study has several strengths. It used a theory-based HBM framework, included a relatively large primary care sample, added split-sample descriptive analyses of the internal clustering of the researcher-developed questionnaire items, reported McDonald’s omega and effect sizes, controlled for major sociodemographic confounders, and applied FDR correction to secondary analyses. Several limitations should nevertheless be considered. First, the cross-sectional design precludes causal inference and permits reverse causation. Second, convenience sampling from one family medicine clinic may have introduced selection bias, and the sample’s high education and public-sector employment profile limits external validity; the results should not be considered nationally representative. Third, all measures were self-reported and may be affected by recall or social-desirability bias. Fourth, prior disaster exposure was not included in the data-collection form. Because previous experience may influence both technology-seeking behavior and preparedness beliefs, residual confounding cannot be excluded. Fifth, the Disaster and Technology Use Questionnaire was developed as a multidomain questionnaire, and its prespecified equal-weighted sum was used as an operational composite index rather than as a validated unidimensional scale score. No formal pre-study content-validity index, external expert-panel assessment, test–retest reliability evaluation, criterion validation, or external replication was conducted. The EFA/CFA findings describe empirical item clustering in this sample and should not be interpreted as full scale validation or as establishing validated subscales. Alternative weighting or domain-specific scoring may produce different estimates. Sixth, the perceived-susceptibility subscale showed borderline internal consistency (α = 0.599; ω = 0.624). Measurement error may attenuate or destabilize associations involving this dimension, so those findings should be interpreted cautiously. Finally, the study measured preparedness beliefs rather than directly observed preparedness behaviors. Although FDR correction reduced the risk of false-positive findings, secondary comparisons remain exploratory.

## 6. Conclusions

Disaster-related digital technology engagement was independently associated with stronger disaster preparedness beliefs among adults attending a primary care clinic in Türkiye, with a modest-to-moderate incremental contribution beyond sociodemographic factors. Associations were observed for perceived susceptibility, perceived benefits, perceived low barriers, cues to action, and self-efficacy, but not for perceived severity. Because the study was cross-sectional, neither causality nor temporal direction can be established. Primary care and public-health initiatives may improve the quality of digital preparedness by promoting official mobile alert systems and disaster applications, teaching emergency-notification and location-sharing functions, supporting secure digital document storage, and providing misinformation-recognition and digital-literacy training. These measures should be paired with community education and accessible non-digital alternatives, particularly for individuals with lower education, income, or digital access. Longitudinal and intervention studies are needed to determine whether such strategies produce measurable improvements in preparedness behavior.

## Figures and Tables

**Table 1:** Sociodemographic Characteristics of the Participants (*n* = 538).

Variable	Category	*n*	%
Age (years)	≤30 years	202	37.5%
	31–50	224	41.6%
	≥51 years	112	20.8%
Gender	Female	300	55.8%
	Male	238	44.2%
Occupation	Retired	38	7.1%
	Unemployed	62	11.5%
	Worker	68	12.6%
	Student	104	19.3%
	Self-employed	36	6.7%
	Civil servant	230	42.8%
Marital Status	Married	306	56.9%
	Single	196	36.4%
	Divorced/Widowed	36	6.7%
Education Level	Primary/Secondary school	60	11.2%
	High school	110	20.4%
	University or higher	368	68.4%
Income Level	Income less than expenses	92	17.1%
	Income equal to expenses	300	55.8%
	Income greater than expenses	146	27.1%

Data are presented as *n* and %.

**Table 2:** Descriptive Statistics and Internal Consistency of the Study Measures.

Variable	Number of Items	Median (IQR)	Cronbach’s α	McDonald’s ω
Questionnaire-Derived Technology Engagement Composite Index	10	36 (30–40)	0.793	0.799
General Disaster Preparedness Belief Scale (Total)	31	114 (103–123)	0.866	0.876
Perceived Susceptibility	6	23 (21–25)	0.599	0.624
Perceived Severity	3	12 (10–13)	0.674	0.719
Perceived Benefits	3	12 (12–15)	0.891	0.893
Perceived Low Barriers	6	20 (17–23)	0.721	0.739
Cues to Action	5	17 (15–20)	0.670	0.690
Self-efficacy	8	29 (26–32)	0.692	0.714

Data are presented as median (interquartile range). IQR: interquartile range. Negatively worded GDPBS items were reverse-coded during data entry; higher perceived-low-barriers scores therefore indicate fewer barriers.

**Table 3:** Split-Sample Exploratory and Confirmatory Analyses of Empirical Item Clustering in the Disaster and Technology Use Questionnaire.

Item	Abbreviated Content	EFA F1	EFA F2	Empirical Domain	CFA Loading
T1	Knowledge of official disaster applications and warning systems	0.635	0.058	F1	0.664
T2	Active use of emergency notifications	0.773	−0.079	F1	0.519
T3	Availability of power bank/radio or similar tools	0.502	0.071	F1	0.463
T4	Importance of digital maps and location sharing	0.234	0.698	F2	0.827
T5	Perceived technical competence to use disaster applications	0.701	0.112	F1	0.574
T6	Concern about technological failure during a disaster	−0.198	0.703	F2	0.466
T7	Belief that technology accelerates post-disaster aid	0.210	0.483	F2	0.562
T8	Expected access to accurate digital information	0.487	0.146	F1	0.474
T9	Digital storage of important documents	0.589	−0.129	F1	0.543
T10	Ability to distinguish accurate from inaccurate information	0.427	0.175	F1	0.426

EFA: *n* = 269, minimum residual extraction with oblimin rotation; CFA: *n* = 269, diagonally weighted least squares. Domain 1 = access, use, and competence; Domain 2 = perceived value and technology-related concerns. KMO = 0.819; Bartlett χ^2^(45) = 1311.50, *p* < 0.001. CFA fit: CFI = 0.952, TLI = 0.936, RMSEA = 0.055. These analyses describe covariance and empirical item clustering and do not establish formal scale validity, unidimensionality, or validated subscales.

**Table 4:** Correlations between disaster-related technology engagement and disaster preparedness beliefs.

Variables	Spearman’s ρ	95% CI	*p*-Value	FDR q-Value
Preparedness Belief (Total)	0.388	0.313 to 0.457	<0.001	Primary analysis
Perceived Susceptibility	0.348	0.272 to 0.420	<0.001	<0.001
Perceived Severity	0.024	−0.060 to 0.109	0.574	0.574
Perceived Benefits	0.323	0.245 to 0.396	<0.001	<0.001
Perceived Low Barriers	0.269	0.189 to 0.346	<0.001	<0.001
Cues to Action	0.317	0.238 to 0.391	<0.001	<0.001
Self-efficacy	0.289	0.209 to 0.364	<0.001	<0.001

Spearman’s rank correlation was used. The total-score association was prespecified as the primary analysis; FDR correction was applied to the six secondary subscale correlations. CI: confidence interval; FDR: false discovery rate.

**Table 5:** Comparison of disaster-related technology engagement and preparedness beliefs by gender.

Variables	Female Median (IQR)	Male Median (IQR)	*p*-Value	FDR q	Rank-Biserial r
Technology Engagement Composite Index	36 (32–39)	35 (29.25–40)	0.396	0.453	0.042
Preparedness Belief Total	115 (105–125)	112 (98–120.75)	0.001	0.001	0.170
Perceived Susceptibility	24 (21–26)	22 (20–25)	<0.001	<0.001	0.178
Perceived Severity	12 (11–14)	11 (9–13)	<0.001	<0.001	0.230
Perceived Benefits	12 (12–15)	13 (12–15)	0.157	0.209	−0.069
Perceived Low Barriers	21 (19–24)	19.5 (16–22)	<0.001	<0.001	0.269
Cues to Action	18 (15–20)	17 (15–19)	0.034	0.055	0.106
Self-efficacy	29 (26–32)	29 (26–33)	0.569	0.569	−0.029

Data are presented as median (interquartile range). Mann–Whitney U tests were used. Positive rank-biserial r indicates higher values among women. FDR correction was applied across the eight gender comparisons.

**Table 6:** Comparison of disaster-related technology engagement and total preparedness belief scores by education.

	Primary/Secondary Median (IQR)	High School Median (IQR)	University or Higher Median (IQR)	*p*-Value	FDR q	ε^2^
Technology Engagement Composite Index	30 (28–35)	35 (26.5–40)	36 (33–40)	<0.001	<0.001	0.065
Preparedness Belief (Total)	105 (93–114.25)	107 (95–115.75)	116 (108–125.25)	<0.001	<0.001	0.103

Data are presented as median (interquartile range). Kruskal–Wallis tests were used. ε^2^ denotes epsilon-squared effect size. FDR-adjusted pairwise Mann–Whitney tests are described in the text.

**Table 7:** Comparison of disaster-related technology engagement and total preparedness belief scores by income level.

	Income ≤ Expenses Median (IQR)	Income > Expenses Median (IQR)	*p*-Value	FDR q	Rank-Biserial r
Technology Engagement Composite Index	35 (30–39.25)	37 (33–40)	0.002	0.002	0.170
Preparedness Belief (Total)	112 (102–121)	119 (110–126)	<0.001	<0.001	0.233

Data are presented as median (interquartile range). Mann–Whitney U tests were used. Participants reporting income below or equal to expenses were combined and compared with those reporting income above expenses. Positive rank-biserial r indicates higher values in the higher-income group.

**Table 8:** Hierarchical multiple linear regression predicting total disaster preparedness belief.

Predictor	B	HC3 SE	95% CI	*p*-Value
Intercept	76.259	7.206	62.135 to 90.383	<0.001
Male (reference: female)	−2.743	1.299	−5.289 to −0.197	0.035
High school (reference: primary/secondary)	1.845	2.528	−3.111 to 6.800	0.466
University or higher (reference: primary/secondary)	9.917	2.581	4.858 to 14.975	<0.001
Income equal to expenses (reference: income below expenses)	3.193	1.789	−0.314 to 6.699	0.074
Income above expenses (reference: income below expenses)	5.275	1.971	1.412 to 9.138	0.007
Single (reference: married)	−2.921	1.721	−6.294 to 0.453	0.090
Divorced/widowed (reference: married)	0.517	2.068	−3.537 to 4.571	0.802
Unemployed (reference: retired)	−0.996	3.534	−7.922 to 5.930	0.778
Worker (reference: retired)	−0.066	3.512	−6.949 to 6.817	0.985
Student (reference: retired)	−0.866	3.572	−7.867 to 6.136	0.809
Self-employed (reference: retired)	−4.212	3.821	−11.702 to 3.277	0.270
Civil servant (reference: retired)	−4.742	2.821	−10.271 to 0.786	0.093
Age (years)	−0.002	0.065	−0.130 to 0.125	0.971
Technology engagement composite index (per 1-point increase)	0.888	0.110	0.672 to 1.103	<0.001

Model 1 included age, gender, education, income, marital status, and occupation (R^2^ = 0.148; adjusted R^2^ = 0.127). Model 2 added the technology-engagement composite index (R^2^ = 0.281; adjusted R^2^ = 0.262; ΔR^2^ = 0.133; F-change = 97.15; *p* < 0.001; Cohen’s f^2^ = 0.186). HC3 heteroscedasticity-robust standard errors are reported.

**Table 9:** Adjusted associations between technology engagement and Health Belief Model subscales.

Outcome	B	95% CI	Standardized β	*p*-Value	FDR q	ΔR^2^
Perceived Susceptibility	0.180	0.132 to 0.229	0.341	<0.001	<0.001	0.099
Perceived Severity	0.027	−0.017 to 0.072	0.068	0.227	0.227	0.004
Perceived Benefits	0.134	0.089 to 0.179	0.366	<0.001	<0.001	0.114
Perceived Low Barriers	0.125	0.057 to 0.192	0.191	<0.001	<0.001	0.031
Cues to Action	0.220	0.169 to 0.271	0.398	<0.001	<0.001	0.134
Self-efficacy	0.201	0.117 to 0.285	0.271	<0.001	<0.001	0.062

Separate hierarchical linear regression models were fitted for each subscale, adjusting for age, gender, education, income, marital status, and occupation. HC3 robust confidence intervals are reported. FDR correction was applied to the six technology coefficients.

## Data Availability

The data presented in this study are available on reasonable request from the corresponding author. The data are not publicly available due to privacy/ethical restrictions.
